# Supplementary material for: Day-to-Day Variability in Measurements of Respiration Using Bioimpedance from a Non-Standard Location
Source: Sensors (Basel). 2024 Jul 16;24(14):4612. doi: 10.3390/s24144612 (PMC11280867; doi:10.3390/s24144612)
Supplement: Supplementary file 1 [file sensors-24-04612-s001.zip › sensors-3056446-supplementary.pdf]

sensors

MDPI

Supplementary Information for

Day-to-Day Variability in Measurements of Respiration Using Bioimpedance from a Non-Standard Location

Krittika Goyal [1], Dishant Shah [1] and Steven W. Day [2,*]

[1] Department of Manufacturing and Mechanical Engineering Technology, Rochester Institute of Technology, Rochester, NY 14623, USA; krgmet@rit.edu (K.G.); ds9091@rit.edu (D.S.)
[2] Department of Biomedical Engineering, Rochester Institute of Technology, Rochester, NY 14623, USA
* Correspondence: Steven.Day@RIT.edu

Table S1. Regression slopes for each replicate (total 4 replicates) depicting relationship between tidal volume and thigh-thigh impedance for each day across five subjects. The last three columns show the mean, SD, and % coefficient of variation (COV) for four replicates on each day.

Subject ID	Day #	n=1	n=2	n=3	n=4	Mean	SD	% COV
Subject 1	Day 1	17.22	16.32	20.69	20.98	18.79	2.37	12
Subject 2	Day 1	25.56	23.81	23.8	28.49	25.41	2.21	9
Subject 3	Day 1	36.28	35.60	41.27	36.22	37.34	2.63	7
Subject 4	Day 1	51.62	40.04	46.80	45.58	46.01	4.75	10
Subject 5	Day 1	38.95	26.03	44.45	34.18	35.90	7.80	22
Subject 1	Day 2	18.72	20.61	14.89	15.11	17.33	2.80	16
Subject 2	Day 2	19.15	16.74	18.83	16.77	17.87	1.29	7
Subject 3	Day 2	26.11	36.86	37.41	31.38	32.94	5.30	16
Subject 4	Day 2	70.42	64.45	76.72	65.88	69.36	5.52	8
Subject 5	Day 2	40.21	46.62	39.18	38.98	41.24	3.62	9
Subject 1	Day 3	15.81	15.73	15.33	15.75	15.65	0.21	1
Subject 2	Day 3	17.92	15.17	16.42	20.12	17.41	2.12	12
Subject 3	Day 3	37.24	42.86	36.12	35.21	37.86	3.43	9
Subject 4	Day 3	72.12	70.38	73.55	81.30	74.34	4.81	6
Subject 5	Day 3	25.89	39.38	46.83	45.30	39.35	9.53	24

Table S2. Regression slopes for each replicate (total 4 replicates), depicting relationship between tidal volume and thorax impedance for each day across five subjects. The last three columns show the mean, SD, and % coefficient of variation (COV) for four replicates on each day.

Subject ID	Day #	n=1	n=2	n=3	n=4	Mean	SD	% COV
Subject 1	Day 1	1.02	1.03	1.18	1.20	1.10	0.09	8
Subject 2	Day 1	2.17	2.85	2.09	2.30	2.35	0.34	15
Subject 3	Day 1	7.51	7.47	7.33	7.91	7.55	0.24	3
Subject 4	Day 1	1.06	1.09	1.13	1.44	1.18	0.17	15
Subject 5	Day 1	4.06	5.75	3.56	3.44	4.20	1.06	25
Subject 1	Day 2	0.74	0.74	0.48	0.49	0.61	0.14	23
Subject 2	Day 2	1.55	1.46	1.41	1.50	1.48	0.06	4
Subject 3	Day 2	10.30	14.79	15.46	13.73	13.57	2.29	17
Subject 4	Day 2	1.15	2.17	2.53	2.85	2.17	0.73	34
Subject 5	Day 2	3.80	3.95	3.75	3.76	3.81	0.09	2
Subject 1	Day 3	0.89	0.89	0.77	0.78	0.83	0.06	8
Subject 2	Day 3	1.46	1.35	1.42	1.69	1.48	0.15	10
Subject 3	Day 3	6.40	6.98	8.07	7.18	7.16	0.69	10
Subject 4	Day 3	7.45	11.75	5.59	5.09	7.47	3.02	41
Subject 5	Day 3	6.37	4.30	5.13	4.29	5.02	0.98	19
